# The Homeless Class in London

**Published:** 1907-08-10

**Authors:** 


### Public Health and Hygiene.

### THE HOMELESS CLASS IN LONDON.

The true anarchist—that is, the person who abides no law—is a natural product of the human family. Among all peoples and in all times he has been found. Whether to-day in our modern cities and among civilised communities he be an example of atavism or not is an open question, but he is a definite measurable product of our population. A recent report by Sir Shirley Murphy submitting a census of the homeless persons in London shows the extent to which he now prevails in the Metropolis. The persons comprised in this census are not the political theorists whose idealism leads them to anticipate the social transcendence of all government; they are, on the contrary, persons probably as devoid of political theories as any which the human race provides. But they are practical anarchists; they resent social restraint, they abhor discipline, they loathe regularity and ordered habit. Hence they choose to "lie rough" in the open streets, to crouch on doorsteps, or huddle together on common staircases and draughty passages rather than avail themselves of the provided accommodation which entails submission to discipline and order. So, at least, must one infer from the figures of the report.

The census was the third organised by the Council, says Sir Shirley Murphy. "The night was clear and cold, and it is therefore somewhat remarkable that fewer than 50 persons were found taking shelter on staircases or in other places affording some protection from the wind. In all, however, the Council's officers counted 1,998 men, 402 women, and 4 children who might be regarded as homeless. Of these, 862 men and 36 women received soup and bread from the Salvation Army on the Embankment, and 156 men were provided with food in the tents of the Church Army. On the same night a census was taken of the number of persons occupying beds in common lodging-houses, with the following result:—

| | Men | Women | Couples |
|---|---|---|---|
| Authorised accommodation ... ... | 25,599 | 2,436½ | 245 |
| Number of persons on night of February 8... ... ... ... ... | 20,438 | 1,598 | 207 |
| Number of vacant beds ... ... ... | 5,161 | 838½ | 33 |
| Number of persons turned away unable to pay, etc. ... ... ... ... | 658 | 21 | 9 |

"On the same night 4,263 men slept in Rowton Houses, which had 844 vacant beds; and 642 men in Bruce House, Kemble Street, where there were 57 vacant beds. It would thus appear that there is more than sufficient accommodation for all the homeless persons in London in existing institutions at an average cost of 6d. per night per head. This is, of course, irrespective of the accommodation provided in casual wards, in which, on the night of February 8, there were 1,137 vagrants. The total accommodation provided in the London casual wards is for 1,882 persons, and there was vacant accommodation, therefore, for 745 persons."

It is clear that the homelessness of these persons is not due wholly to lack of accommodation, nor to any utter inability to avail themselves of it, since on the night in question the London casualty wards had vacant accommodation for over 700 persons. Social deterioration, in fact, has not reached bottom at the level of the pauper vagrant. Possibly it would do, but that the further stage—the reversion to homelessness—is widely fostered by well-meaning but unscientific attempts to ameliorate the condition of these decadent objects of human sympathy.

"It is interesting to note," says the report, "the facilities afforded in London for obtaining cheap or free food. At a large mission hall in Shoreditch about 600 men are fed daily at a very small cost, a substantial meal being provided for 2d. or 3d. a head. Here, too, free food is distributed to 350 men each Sunday and to 900 men each Thursday. On the Embankment each night from 900 to 1,500 persons are supplied with a meal of soup and bread free of charge. There is, moreover, an institution in Limehouse which sends a man out each night to different parts of London to distribute tickets in the streets to the homeless poor. Each ticket entitles a man to eight ounces of bread, and some 400 tickets are distributed each evening. The distribution of soup and bread on the Embankment begins at 1 A.M., and the tickets for bread in the Limehouse institution are available from midnight to 4 A.M. It is, therefore, somewhat significant that on the morning of February 9 no applications were received at the Limehouse institution until 2.15 A.M., though from that time until 4 A.M. 277 tickets were presented in exchange for bread. From the information gathered it would appear that it is possible for a man to live, so far as food is concerned, for a penny a day, provided he takes advantage of the free food distribution and supplements this by purchasing food at the low rates which prevail in the Mission Hall, Shoreditch.

The effect of the relief thus afforded is to make the choice between the last social discipline with shelter, and utter anarchy without it, possible. The human family, it is to be presumed, will continue to produce the type of which the homeless are examples, but were our treatment of them more tonic their degradation would be less complete, and they would in lesser degree be a menace to civic well-being. The Public Health Committee of the London County Council appear to have arrived at conclusions in harmony with this view. They say:—

"The Committee refer to the danger to the community that the sleeping-out class constitute by the spread of vermin and disease; they point out that the man who is homeless can obtain clean accommodation and wholesome food in the casual ward, and state that, if he refuses to use the ward, the law should be applied both to defend the community and to protect the man himself. They conclude that the enforcement of measures against the sleeping-out class would rapidly reduce it, and would deter others from sinking into it. They suggest amendment of the law so as to make sleeping out an offence whenever it is a danger or nuisance to the public."

